# The Footprints of Poly-Autoimmunity: Evidence for Common Biological Factors Involved in Multiple Sclerosis and Hashimoto’s Thyroiditis

**DOI:** 10.3389/fimmu.2018.00311

**Published:** 2018-02-20

**Authors:** Simona Perga, Serena Martire, Francesca Montarolo, Ilaria Giordani, Michela Spadaro, Gabriele Bono, Stefania Corvisieri, Ilaria Messuti, Giancarlo Panzica, Fabio Orlandi, Antonio Bertolotto

**Affiliations:** ^1^Neuroscience Institute Cavalieri Ottolenghi (NICO), Orbassano, Turin, Italy; ^2^Regional Reference Centre for Multiple Sclerosis (CReSM), University Hospital S. Luigi Gonzaga, Orbassano, Turin, Italy; ^3^Department of Neuroscience Rita Levi Montalcini, University of Turin, Turin, Italy; ^4^SCDU Endocrinology and Metabolism, Humanitas Gradenigo Hospital, Department of Oncology, University of Turin, Turin, Italy

**Keywords:** multiple sclerosis, Hashimoto’s thyroiditis, gene expression, 25-hydroxy vitamin D, regulatory T cells

## Abstract

Autoimmune diseases are a diverse group of chronic disorders and affect a multitude of organs and systems. However, the existence of common pathophysiological mechanisms is hypothesized and reports of shared risk are emerging as well. In this regard, patients with multiple sclerosis (MS) have been shown to have an increased susceptibility to develop chronic autoimmune thyroid diseases, in particular Hashimoto’s thyroiditis (HT), suggesting an autoimmune predisposition. However, studies comparing such different pathologies of autoimmune origin are still missing till date. In the present study, we sought to investigate mechanisms which may lead to the frequent coexistence of MS and HT by analyzing several factors related to the pathogenesis of MS and HT in patients affected by one or both diseases, as well as in healthy donors. In particular, we analyzed peripheral blood mononuclear cell gene-expression levels of common candidate genes such as TNFAIP3, NR4A family, BACH2, FOXP3, and PDCD5, in addition to the regulatory T cell (Treg) percentage and the 25-hydroxy vitamin D serum levels. Our findings support the plausibility of the existence of common deregulated mechanisms shared by MS and HT, such as BACH2/PDCD5-FOXP3 pathways and Tregs. Although the biological implications of these data need to be further investigated, we have highlighted the relevance of studies comparing different autoimmune pathologies for the understanding of the core concepts of autoimmunity.

## Introduction

In recent years, there has been an extensive search for pathophysiological mechanisms that may underlie autoimmunity resulting in the frequent co-occurrence of autoimmune disorders (ADs) such as multiple sclerosis (MS) and Hashimoto’s thyroiditis (HT) among others (1–5). MS is the most common AD of the central nervous system (CNS) involving deregulated immune activation (6). HT, characterized by antithyroid autoantibodies in the serum, such as antithyroglobulin (Tg) and antithyroperoxidase (TPO), along with lymphocyte infiltration of the gland, is the most frequent endocrine disease and the most common autoimmune thyroid disease (7, 8). Several studies have suggested genetic, epigenetic, environmental, and infectious agents as interacting factors influencing the risk for the development of these disorders. Nevertheless, the etiology of both these diseases is still largely unknown and the pathological mechanisms responsible for their co-occurrence remain poorly understood.

There have been reports hypothesizing shared risk (9, 10) and increased statistical susceptibility for people with one AD to develop another, suggesting the concept of an autoimmune predisposition (10–12). In the present study, we sought to investigate the underlying mechanisms that may lead to this phenomenon of poly-autoimmunity in patients with MS and HT.

Genome-wide association studies (GWAS) have identified hundreds of risk loci for autoimmunity till date (13). Although most of the associations have been linked to classical HLA alleles, non-HLA genes have also been demonstrated to contribute to AD susceptibility (13). Among these, genetic variants involved in the regulation of NF-kB pathway such as TNFAIP3 and NR42A are prominent risk factors for MS. Enhanced NF-kB activation and greater responsiveness to inflammatory stimuli is an important aspect of MS pathology wherein these genes may play a role (14, 15). Previous studies by our group have demonstrated a marked decrease in gene-expression levels of both these NF-kB inhibitors in whole blood and peripheral blood mononuclear cells (PBMCs) of MS patients compared with healthy controls (HCs) (16–19). Polymorphisms in the TNFAIP3 locus are associated with other ADs as well (20) and cell-specific TNFAIP3 deletion in mice triggers inflammatory phenotypes mimicking those seen in the genetically associated ADs (21). The other candidate gene, the nuclear receptor NR4A2, together with the other members of the NR4A family (NR4A1 and NR4A2), promotes the expression of the transcription factor FOXP3 and the generation of regulatory T cells (Tregs) (22, 23). The deletion of NR4A in mice causes massive multi-organ inflammation, partially regenerating the phenotype observed in human ADs (22).

Apart from these disease-specific risk factors, there are common genetic loci shared between MS and other ADs including HT (14, 24). The Treg-modulating transcription factor BACH2 is one such common gene that stabilizes Treg development and is essential for suppression of lethal inflammation (25). Although BACH2 has not been shown to be directly involved in NF-kB pathway, its expression is regulated by the NF-kB subunit c-Rel (26). Notably, BACH2 transcript levels are decreased in whole blood of MS patients compared with HC (27).

In contrast to the wealth of information available from GWAS related to MS, specific GWAS conducted on large cohorts of patients with HT are lacking. Nevertheless, GWAS in patients with autoimmune thyroid disease or hypothyroidism have been undertaken that provide insights into the disease (28). Interestingly, these studies show certain common candidate genes that maybe shared between HT and MS. Susceptibility to HT has been associated with single-nucleotide polymorphisms in the NF-kB subunit NFKB1 (29). Also, TNFAIP3 has been detected among the susceptibility loci for Graves’ Disease, an autoimmune thyroid disease characterized by hyperthyroidism (30, 31). Moreover, BACH2 has been described as an autoimmune thyroid disease-associated region (24), together with another key gene involved in the development of Tregs, such as FOXP3 (32, 33).

Treg modulation is important in the pathogenesis of both MS and HT. As suggested by experimental evidences and genetic associations, most autoimmune conditions are characterized by an altered balance between effector and Tregs, with the latter having lost the ability to maintain immune tolerance to self-antigens (34). The molecular mechanisms involved in the development and maintenance of Tregs have been extensively investigated and several genes controlling their differentiation and function have been identified. Reduced number and/or function of Tregs have been observed in both MS and HT (35–44), although their precise role is still matter of debate. However, the deregulation of some of the genes involved in Treg modulation, such as NR4A2, BACH2, FOXP3, and PDCD5, have been directly associated with the pathogenesis of MS or HT (14, 16–18, 24, 27, 45–49).

Here, insufficient vitamin D levels have been shown to contribute to Treg modulation and AD susceptibility as well. Several studies demonstrate that vitamin D modulates cell-mediated immune responses, suppressing inflammatory T-cell activity, and promoting Treg induction (50–52). Furthermore, mounting epidemiological evidence suggests an association between vitamin D deficiency and a higher incidence of autoimmune diseases, including MS and HT (53).

Despite this background, a thorough understanding of the pathophysiological mechanisms that may underline poly-autoimmunity is still missing. In the present study, we investigated factors demonstrated to be involved in the development of MS and HT that are frequently coexisting ADs. In particular, we analyzed PBMC gene-expression levels of common candidate genes such as TNFAIP3, NR4A family, BACH2, FOXP3, and PDCD5, in addition to the Treg percentage and the 25-hydroxy (25-OH) vitamin D serum levels. To the best of our knowledge, this is the first study directly comparing these two pathologies that may contribute to the understanding of poly-autoimmunity. Here, we sought to investigate if differential breakdown of similar anti-inflammatory mechanisms could contribute to the distinct levels of inflammation seen in patients with MS, HT, or both. This data may also explain if having a greater deregulation puts the patient at a higher risk for developing poly-autoimmunity.

## Materials and Methods

### Patients and Controls

This study was carried out in accordance with the recommendations of the Declaration of Helsinki. The protocol was approved by the local Ethics Committee of University Hospital San Luigi Gonzaga, Piedmont region (18/01/2013 N.20). All subjects gave written informed consent.

For this study, 55 treatment-naïve patients diagnosed with HT were selected among patients followed up at the Struttura Complessa of Endocrinology of the Presidio Sanitario Gradenigo, Turin. The diagnosis of HT was confirmed by conventional clinical, laboratory, and ultrasonographic findings and was defined by the presence of at least one type of antithyroid antibody among anti-TPO and anti-TG. HT patients were classified, according to the normal laboratory reference values of serum thyroid-stimulating hormone (TSH), in 34 euthyroid (HTE) (TSH from 0.5 to 4.5 mcU/mL) and 21 hypothyroid (HTI) (TSH > 4.5 mcU/mL) patients.

Samples from 59 treatment-naïve patients with a diagnosis of relapsing–remitting (RR) MS, according to the revised McDonald criteria (54), were provided by the Regional Reference Centre for MS (CReSM) Bio-bank, San Luigi University Hospital, located at the Neuroscience Institute Cavalieri Ottolenghi, Orbassano. These patients did not suffer any exacerbations or received corticosteroids during the month before sampling. They were negative for the presence of antithyroid antibodies and had normal TSH values.

A third group of 13 treatment-naïve RRMS patients diagnosed with the co-occurrence of HT (MS + HT) simultaneously according to the criteria described above was selected.

Finally, 59 HCs were enrolled as a control group by the two centers. They were negative for the presence of antithyroid antibodies and had normal TSH values and ultrasonographic analysis. Moreover, they had no family history of MS or HT in their first-degree relatives.

The following inclusion criteria were adopted for all the groups: (1) no signs of acute infection or illness during the month prior to blood collection and (2) absence of chronic comorbidities.

Thyroid ultrasonography was performed by an experienced radiologist, using a 7-MHz linear and an SSA 770 Aplio scanner (Toshiba Medical SystemsCo, Ltd., Tokyo, Japan).

Detailed demographic and clinical features are reported in Table 1.

**Table 1:** Demographical and clinical characteristics.

	HC (*n* = 59)	HTE (*n* = 34)	HTI (*n* = 21)	HT (*n* = 55)	MS (*n* = 59)	MS-HT (*n* = 13)	*p*-value
Female, *n* (%)	45 (76)	27 (79)	17 (81)	44 (80)	40 (68)	11 (85)	ns
Age, median (range)	42 (22–79)	43.5 (21–75)	51 (23–72)	47 (21–75)	35 (15–65)	39 (27–58)	0.001
TSH, median (range)	1.41 (0.51–4.60)	1.99 (0.62–4.30)	6.6 (4.65–62.30)	3.73 (0.62–62.30)	1.43 (0.43–4.07)	1.74 (0.02–5.47)	
AbTPO, median (range)	0.4 (0.1–16.3)	168.2 (0.4–5,677)	448 (0.2–6,500)	244 (0.2–6,500)	0.4 (0.0–9.2)	90.1 (20.1–1,266.9)	
AbTG, median (range)	0.3 (0–7.8)	22.5 (0–7,537)	50 (0.3–2,462)	35 (0–7,537)	0.3 (0–4.9)	2.7 (0.3–59.9)	
Disease duration, months, median (range)					26 (1–235)	27 (1–235)	ns
No. of relapses the year before, median (range)					1 (0–2)	1 (0–2)	ns
EDSS score, median (range)					1 (0–6)	1 (0–6)	ns

*HC, healthy controls; HTE, euthyroid patients with Hashimoto’s thyroiditis; HTI, hypothyroid patients with Hashimoto’s thyroiditis; HT, patients with Hashimoto’s thyroiditis; MS, patients with multiple sclerosis; MS-HT, patients affected by both multiple sclerosis and Hashimoto’s thyroiditis; TSH, thyroid-stimulating hormone; AbTPO, antithyroperoxidase antibodies; AbTG, antithyroglobulin antibodies; EDSS, Expanded Disability Status Scale*.

### Sample Collection

Blood collection was done in the morning between 8:30 and 10:30 a.m. For each subject, 6 mL of whole blood was collected in Vacutainers with serum separator and 24 mL in EDTA Vacutainers (BD Biosciences, Milan, Italy). After collection, samples were immediately processed as follows:

Serum samples were centrifuged for 10 min at 3,000 rcf, aliquoted, and stored at −80°C until use. PBMCs were freshly isolated by Lymphoprep density-gradient centrifugation according to the manufacturer’s instructions. Cells were split in two aliquots, both stored at −80°C until use: one was resuspended in RNA-Later solution (Thermo Fisher Scientific, Waltham, MA, USA) for total RNA extraction and the other was stored in a cryopreserving solution containing 30% FBS (Thermo Fisher Scientific), 10% DMSO (Sigma-Aldrich, St Louis, MO), and 60% RPMI (Thermo Fisher Scientific) for flow-cytometry analysis. Whole blood for total RNA extraction was resuspended in homogenization buffer (Promega, Monza, Italy) and stored at −80°C until use.

### Serum Examination

Serum levels of TSH, anti-TPO, and anti-TG antibodies and 25- OH vitamin D were determined at the Analysis Laboratory of Gradenigo Hospital in a single analytical session. Determinations of TSH levels and antibodies titer were performed by two-site quantitative automated immunoenzymatic assays using the following kits: Access HYPERsensitive hTSH, Access Thyroglobulin Antibody II, and Access Thyroperoxidase Ab (Beckman Coulter). The 25-OH vitamin D levels were determined in all the enrolled subjects by chemiluminescence immunoassay using the LIAISON 25-OH Vitamin D TOTAL Assay (DiaSorin, Saluggia, Italy).

### RNA Extraction and Gene-Expression Analysis

Gene-expression analyses were conducted on 34 HTE, 20 HTI, 22 MS, 7 MS + HT, and 56 HC. Total RNA was extracted using the automated Maxwell^®^ Rapid Sample Concentrator (RSC) Instrument, with Maxwell^®^ 16 LEV simplyRNA Blood Kit (Promega) for whole blood and simplyRNA Tissue kit (Promega) for PBMCs, following the manufacturer’s instructions. Total RNA was reverse-transcribed at a final concentration of 20 ng/µL using High-Capacity cDNA Reverse Transcription Kit (Thermo Fisher Scientific). Gene-expression analysis was performed by real-time PCR using TaqMan^®^ gene-expression products (Thermo Fisher Scientific). Expression levels of target genes were calculated by the normalized comparative cycle threshold (*Ct*) method (2^−ΔΔCt^), using glyceraldehyde-3-phosphate dehydrogenase (GAPDH) as reference gene and the Universal Human Reference RNA (Stratagene, Santa Clara, CA, USA) as calibrator.

### Flow Cytometry

Regulatory T-cell levels were evaluated in 29 HTE, 17 HTI, 22 MS, 12 MS + HT, and 33 HC. After gentle thawing at 37°C, PBMCs were immediately added to 5-mL RPMI 1640 (Thermo Fisher Scientific), supplemented with 10% FBS (Thermo Fisher Scientific) and centrifuged to remove DMSO (Sigma-Aldrich). Samples were resuspended in RPMI 1640 medium supplemented with 10% heat-inactivated FBS and counted for flow-cytometry experiments.

For Treg evaluation, PBMCs were incubated for 5 min at 4°C with rabbit immunoglobulins G (IgG, Sigma-Aldrich) to block non-specific sites and then for 10 min at 4°C with fluorochrome-conjugated monoclonal Ab (mAb) or isotype-matched negative controls. For Treg determination the following antihuman mAbs were used: CD4 PE-Vio770, CD25 APC, and CD127 FITC (Miltenyi Biotec, Bergisch Gladbach, Germany). Living cells identified by propidium iodide (Sigma-Aldrich) exclusion were gated according to their light-scatter properties to exclude cell debris. Samples were analyzed using a CyAn ADP, running Summit 4.3 software (Beckman Coulter, Brea, CA, USA).

### Statistical Analysis

Continuous data are presented as medians and ranges and discrete data are given as counts and percentages. Fisher’s exact tests were performed to compare groups of categorical data. Mann–Whitney *U* test and Kruskal–Wallis with Dunn’s posttest with false discovery rate (FDR) correction method were used to compare medians between groups of continuous data, as appropriate. The correlation between gene expression, vitamin D, Treg levels, and clinical and demographical variables was assessed by Pearson’s or Spearman’s correlations, as appropriate. These variables have been described in Table 1: (1) sex and age at sampling, TSH, anti-TPO, and anti-TG Ab levels for all the groups and (2) disease duration, number of relapses 1 year before sampling and EDSS score at sampling for MS and MS + HT groups. Statistical significance was considered at *p* ≤ 0.05. All analyses were carried out using R version 3.1.1 (www.r-project.org).

## Results

Demographic and clinical data of all subjects are summarized in Table 1. There was no difference observed in the gender distribution between groups. The median age of the MS group was significantly lower compared with HC, the, and HTI (Dunn’s posttest with FDR correction, *p* = 0.03, *p* = 0.01, and *p* = 0.002, respectively) which is likely due to the difference in the age of onset of the two diseases (6, 8).

Notably, the median disease duration of MS and MS + HT patients (defined as the time from the onset of the first disease symptoms to blood sampling) was just over 2 years, because patients were selected among those who had just received the MS diagnosis and had not yet started disease-specific therapies.

### Differential Expression Levels of Candidate Genes among Study Groups

To investigate the deregulated mechanisms possibly shared by MS and HT, we measured the PBMC gene-expression levels of a panel of key molecules (i.e., TNFAIP3, NR4A family, BACH2, PDCD5, and FOXP3) in all study groups through real time PCR. Notably, the gene expression of the whole panel was first compared between HTE and HTI. If no differences were revealed, they were considered as a single HT group for subsequent analyses for that particular gene.

**Figure 1:**
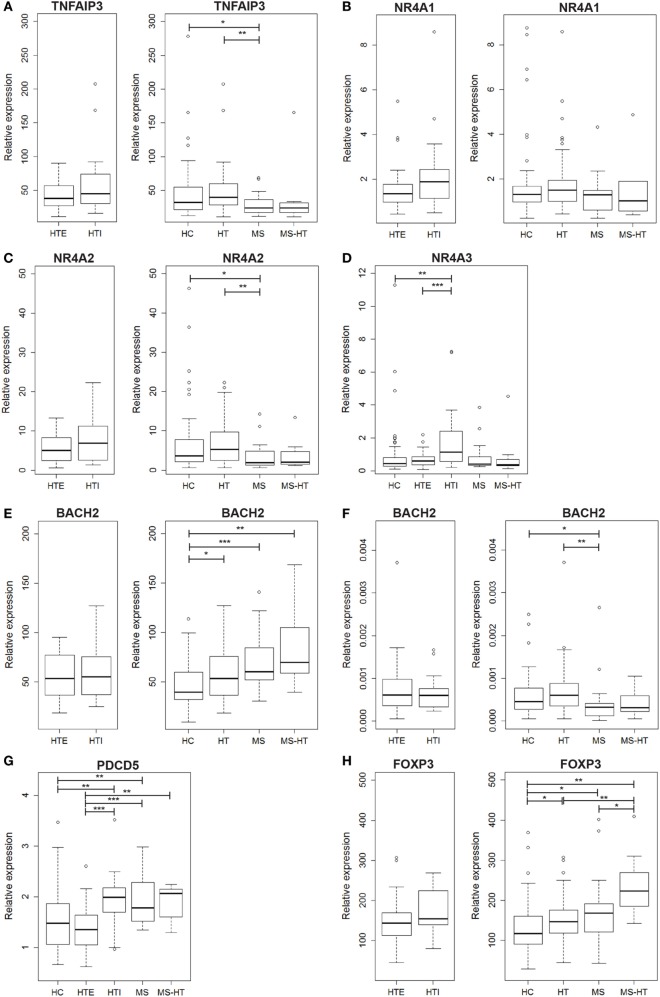
Peripheral blood mononuclear cell (PBMC) gene-expression levels of **(A)** TNFAIP3, **(B)** NR4A1, **(C)** NR4A2, **(D)** NR4A3, **(E)** BACH2, **(G)** PDCD5, and **(H)** FOXP3, and **(F)** whole peripheral blood gene-expression level of BACH2. Relative expression was calculated by the normalized comparative cycle threshold method (2^−ΔΔCt^) and differences between groups were evaluated by the Mann–Whitney *U* test.

We found that TNFAIP3 gene expression was lower in MS compared with both HC and HT groups (Mann–Whitney *U* test, *p* = 0.04 and *p* = 0.005, respectively). A similar downregulation was found also for MS + HT patients, but it did not reach statistical significance (Figure 1A, right panel). On the contrary, HT patients did not show a downregulated gene expression. No significant differences were found between HTE and HTI (Figure 1A, left panel).

In the NR4A family, NR4A1 transcript level was similar among all groups (Figure 1B), while NR4A2 was underexpressed in MS compared with both HC and HT (Mann–Whitney *U* test, *p* = 0.03 and *p* = 0.002, respectively) (Figure 1C, right panel), corroborating previous studies (17, 18). NR4A3, however, was expressed at a higher level in HTI compared with HTE and HC (Mann–Whitney *U* test, *p* = 0.0006 and *p* = 0.001, respectively) (Figure 1D). Notably, a decreased expression of both genes was also found in MS + HT patients compared with HC.

The next candidate gene, BACH2, was overexpressed in HT, MS, and MS + HT compared with HC (Mann–Whitney *U* test, *p* = 0.01, *p* = 0.0006, and *p* = 0.005, respectively) (Figure 1E, right panel). No significant differences were found between HTE and HTI forms (Figure 1E, left panel). These data are in contrast with previous results obtained in our laboratory on whole blood, demonstrating a downregulation of BACH2 gene expression in therapy-naïve MS patients as compared with HC (27). To reproduce the same experimental conditions as before, we repeated the measurement on whole blood obtained from the same individuals enrolled in the study. In this case, BACH2 was underexpressed in MS compared with both HC and HT (Mann–Whitney *U* test, *p* = 0.04 and *p* = 0.002, respectively) (Figure 1F, right panel), confirming previous data.

On the other hand, PDCD5 gene was found to be differently expressed in HTE and HTI groups. HTI and MS patients showed higher PDCD5 levels compared with both HC (Mann–Whitney *U* test, *p* = 0.005, and *p* = 0.006, respectively) and HTE (Mann–Whitney *U* test, *p* = 0.0003 and *p* = 0.0001, respectively). The gene was expressed at higher levels also in MS + HT patients compared with HTE (Mann–Whitney *U* test, *p* = 0.008) and HC, although the latter did not achieve statistical significance (Mann–Whitney *U* test, *p* = 0.06) (Figure 1G).

**Figure 2:**
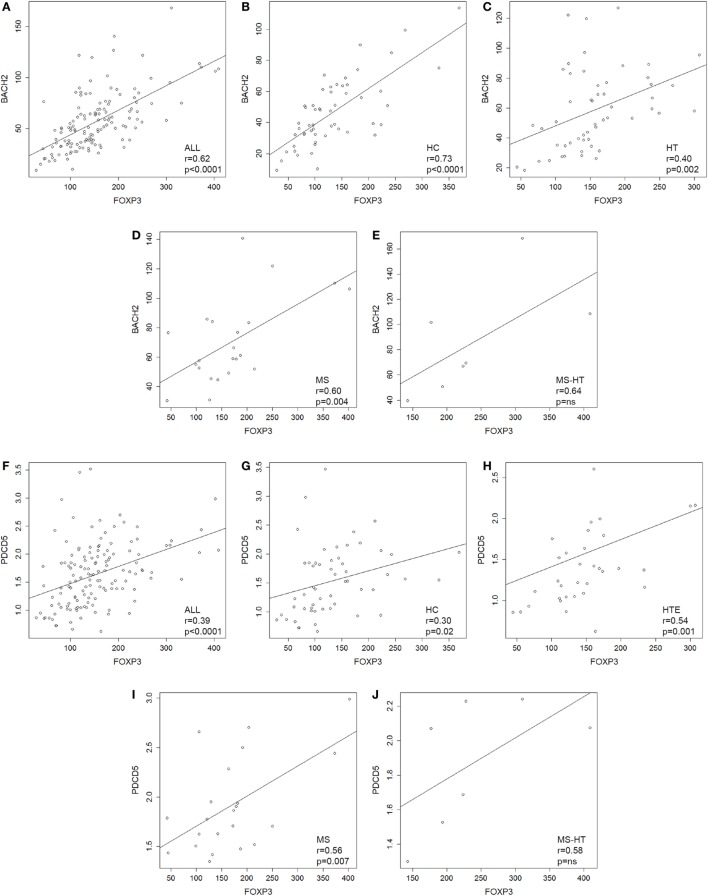
**(A–E)** Scatterplots showing the relationship between peripheral blood mononuclear cell (PBMC) gene-expression levels of BACH2 and FOXP3 in **(A)** the whole cohort of individuals and in **(B)** healthy control (HC), **(C)** Hashimoto’s thyroiditis (HT), **(D)** multiple sclerosis (MS), and **(E)** MS + HT separately. **(F–J)** Scatterplots showing the relationship between PBMCs gene-expression levels of PDCD5 and FOXP3 in **(F)** the whole cohort of individuals and in **(G)** HC, **(H)** HTE, **(I)** MS, and **(J)** MS + HT separately. Linear correlation was evaluated by Pearson’s correlation analysis.

FOXP3 gene was overexpressed in HT, MS, and MS + HT compared with HC (Mann–Whitney *U* test, *p* = 0.01, *p* = 0.04, and *p* = 0.002, respectively), with the MS + HT group expressing the highest levels (Mann–Whitney *U* test, *p* = 0.008 and *p* = 0.03 for comparisons with HT and MS, respectively) (Figure 1H, right panel). No significant differences were found between HTE and HTI, although FOXP3 levels in the HTI group appeared to be higher (Figure 1H, left panel). A significant correlation between BACH2 and FOXP3 levels was found in the overall population and in HC, HT, and MS separately (Pearson’s correlation, *r* = 0.62 and *p* < 0.0001 for the overall population; *r* = 0.73 and *p* < 0.0001 for HC; *r* = 0.40 and *p* = 0.002 for HT; *r* = 0.60 and *p* = 0.004 for MS) (Figures 2A–E). Similarly, a significant, although weaker, correlation between PDCD5 and FOXP3 levels was found in the overall population and in HC, HT, and MS separately (Pearson’s correlation, *r* = 0.39 and *p* < 0.0001 for the overall population; *r* = 0.30 and *p* = 0.02 for HC; *r* = 0.34 and *p* = 0.01 for HT; *r* = 0.56 and *p* = 0.007 for MS). In particular, this correlation was found in HTE (Pearson’s correlation, *r* = 0.54 and *p* = 0.001) (Figures 2F–J) but not in HTI (data not shown).

### Treg Deficiency Affecting Multiple Sclerosis (MS) Patients to a Greater Degree as Compared with Hashimoto’s Thyroiditis

**Figure 3:**
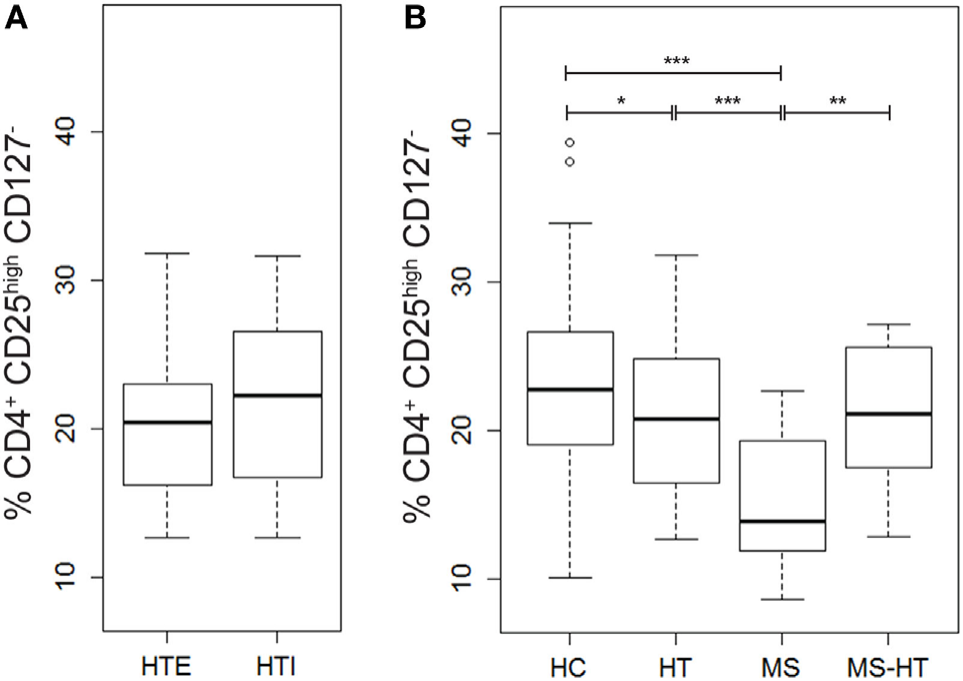
Percentage of Tregs, defined as CD4^+^CD25^high^CD127^−^ cells, in **(A)** hypothyroid (HTI) and euthyroid patients with Hashimoto’s thyroiditis (HTE) separately and in **(B)** healthy control (HC), Hashimoto’s thyroiditis (HT), multiple sclerosis (MS), and MS + HT. Differences between groups were evaluated by the Mann–Whitney *U* test.

In order to evaluate possible deregulation of Treg number in the patient groups of this study, the percentage of this key regulatory cell population (identified as cells marked by CD4^+^CD25^high^CD127^−^ as proposed by the Immunophenotyping Consortium) (55) was measured in PBMCs by flow-cytometry analysis. We observed reduced Treg levels in the MS group compared with all other groups (Mann–Whitney *U* test, *p* < 0.0001, *p* = 0.002, and *p* < 0.0001 for HT, MS + HT, and HC, respectively) and in the HT group compared with HC (Mann–Whitney *U* test, *p* = 0.04) (Figure 3B). No differences were found between HTE and HTI (Figure 3A).

No correlations between gene-expression levels and Treg percentage were found (data not shown).

### 25-OH Vitamin D Levels Significantly Reduced in MS Patients

**Figure 4:**
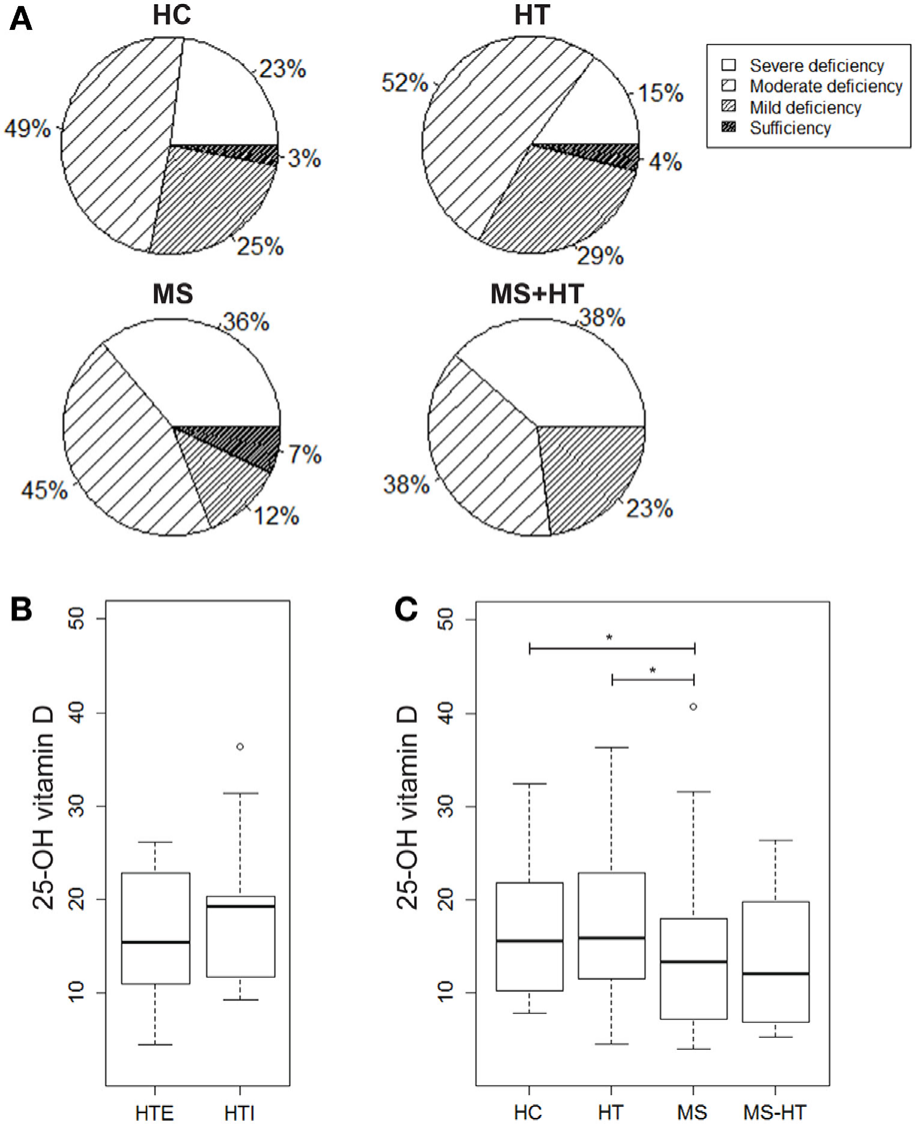
**(A)** Percentage of individuals for each group showing severe 25-OH vitamin D deficiency (<10 ng/mL), moderate deficiency (10–19.9 ng/mL), mild deficiency (20–29.9 ng/mL), or sufficiency (>30 ng/mL). 25-OH vitamin D serum levels in **(B)** hypothyroid (HTI) and HTE separately and in **(C)** healthy control (HC), Hashimoto’s thyroiditis (HT), multiple sclerosis (MS), and MS + HT. Differences between groups were evaluated by the Mann–Whitney *U* test.

In order to evaluate a possible association of vitamin D levels and the ADs analyzed here, serum levels of the active metabolite 25-OH vitamin D were measured in samples from HC, HT, MS, and MS + HT groups. Serum 25-OH vitamin D levels were low overall, as only 4% of our study population (two HC, four MS, and two HT) reached the sufficiency threshold of ≥30 ng/mL. In particular, 23% of HC, 15% of HT, 36% of MS, and 38.5% of MS + HT showed a severe deficiency, defined as <10 ng/mL; 49% of HC, 52% of HT, 45% of MS, and 38.5% of MS + HT showed a moderate deficiency, defined as 10–19.9 ng/mL; 25% of HC, 29% of HT, 12% of MS, and 23% of MS + HT showed a mild deficiency, defined as 20–29.9 ng/mL (Figure 4A). Fisher’s exact test did not highlight significant differences between expected and observed frequencies for 25-OH vitamin D levels categories within groups.

25-Hydroxy vitamin D levels were significantly lower in MS compared with both HC and HT (Mann–Whitney *U* test, *p* = 0.04 and *p* = 0.02, respectively). Also, in the MS + HT group, the 25-OH vitamin D levels seemed to be lower as compared with HC and HT, but the difference did not reach statistical significance (Figure 4C). No difference was found between HTE and HTI (Figure 4B) and no correlation between 25-OH vitamin D and Treg or gene-expression levels was highlighted (data not shown).

## Discussion

Out of the many different comorbidities that may be observed with MS, HT, together with psoriasis, is the most commonly reported autoimmune condition in patients and their relatives (1–3).

The main purpose of this study was to investigate the possible common molecular mechanisms underlying MS and HT. Additionally, we tried to understand whether a high “degree” of autoimmunity, such as that observed in MS patients also affected by HT, could be related to a more profound alteration of these shared pathological mechanisms. A better understanding of these altered mechanisms could provide important insights into the etiology and pathogenesis of these disorders along with unraveling potential therapeutic targets.

The shared risk of these diseases may reflect common genetic susceptibility and/or environmental exposures. Thus, we analyzed several factors that are known to be associated with the pathogenesis of both disorders in patients affected by MS, HT, or both.

Notably, this study has strict inclusion criteria to only consider patients who are at the beginning of their illness and have not yet started specific pharmacological treatment. Therefore, the size of the MS + HT group was limited by the difficulty to find patients who had already developed HT at the time of MS diagnosis, given the different age of onset of the two pathologies. The control group has been selected carefully as well. Only individuals who are negative for antithyroid antibodies, free of any acute or chronic illness and with no family history of any of the two diseases have been included in the HC. Although it is significantly difficult to obtain such ideal cohorts, it is crucial to ensure optimum conditions for studying any immunological phenomena, free from the influence of therapies. The HT group was further divided in euthyroid (HTE) and hypothyroid (HTI) patients according to their TSH level, to investigate potential differences between patients with subclinical and more severe forms of the disease.

First, we measured the PBMC expression levels of a panel of genes that play a key role in the immune system regulation and whose altered expression or function have been associated with the pathogenesis of one or both the disorders. This panel included candidate genes such as TNFAIP3, the NR4A family, BACH2, PDCD5, and FOXP3.

TNFAIP3 and the NR4A family, including NR4A1, NR4A2, and NR4A3, represent a group of potent inhibitors of NF-kB pathway (56, 57), whose deregulation has been associated to several chronic inflammatory and autoimmune conditions including MS (58, 59). Here, we demonstrate that both TNFAIP3 and NR4A2 show decreased expression levels in MS patients compared with the HC group. These data corroborate previous findings by our group obtained on independent cohorts of HC and MS (16–19). Conversely, our study did not show an altered expression of either TNFAIP3 or NR4A2 in the HT group, suggesting that the deregulation of these two NF-kB inhibitors might be an MS-specific pathogenic mechanism. Although it did not reach statistical significance, a decreased expression of both genes was also found in MS + HT patients compared with HC, suggesting a major contribution of the MS disease in these patients.

The other two members of the NR4A family, namely NR4A1 and NR4A3, share a high degree of homology in the molecular structure, as well as complementary functions with NR4A2 (60). However, contrary to NR4A2, the gene-expression levels of these two nuclear receptors did not show differences between groups, except for NR4A3 which was expressed at a higher level in HTI compared with HTE and HC. These data may suggest a specific deregulation mechanism for NR4A2 in MS and a particular function of this gene, which is not compensated by the other members of the family.

Similar to TNFAIP3 and the NR4A family, BACH2, PDCD5 and FOXP3 are among the NF-kB target genes (26, 61, 62). Furthermore, BACH2 and PDCD5 have been demonstrated to be directly involved in the upregulation of FOXP3 gene expression and the enhancement of its function, similar to the NR4A family (23, 63, 64). Our results indicate that BACH2 and FOXP3 are upregulated in MS, HT, and MS + HT compared with HC, with the MS + HT group expressing the highest levels. PDCD5 is upregulated as well in all patient groups in comparison to HC, with the exception of HTE patients. In addition, a significant correlation of FOXP3 levels with BACH2 and with PDCD5 levels was found in HC, MS, and HT groups individually. These data suggest that BACH2 and PDCD5 both may play a role to induce FOXP3 expression in PBMCs in the two ADs. Given that BACH2, PDCD5, and FOXP3 are immuno-modulating molecules with a major anti-inflammatory and immunosuppressive role (25, 63, 65), an increase in their expression levels in both the ADs may appear unexpected. However, this could be explained by considering the broad spectrum of action of these molecules, as described below.

Our findings show that BACH2 is expressed at different levels in whole blood and PBMCs, suggesting that it could play different cell-specific roles. For example, BACH2 is essential for the development of effector-memory and Tregs (25, 66), B cells (67, 68) and alveolar macrophages (69). In addition, new evidences suggest its involvement in the homeostasis of granulocytes. In particular, a deregulation of BACH2 expression in lymphocytes has been proposed to alter the granulocytes balance (70), but there are no indications of how it could affect their gene expression in the literature.

PDCD5 exerts complex biological functions, some of which have not yet been defined. It can accelerate apoptosis in different types of cells in response to various stimuli and control autoimmunity *via* the FOXP3–Tregs axis (61, 63). PDCD5 transcription and protein activity is enhanced by the NF-kB p65 subunit (61). Its abnormal expression has been shown to be involved in autoimmune diseases and inflammatory processes. For example, similar to our results, PDCD5 levels in serum and synovial fluid of rheumatoid arthritis (RA) patients have been found to be significantly higher compared with HC, and a negative correlation of its levels with disease activity indices has been described (71). PDCD5 transcript levels were also found to be elevated in the monocytes activated by inflammatory stimuli (72), in HIV-infected individuals (73) and in patients with chronic liver diseases (74). Furthermore, apoptosis is one of the processes used by organisms to counteract the aberrant survival of autoreactive lymphocytes (75) and is crucial in the pathogenesis and the development of hypothyroidism (7). In this context, our findings suggest that elevated PDCD5 levels in MS and HT could represent a defense mechanism aimed at eliminating auto-reactive immune cells characterized by an “apoptosis-resistant” behavior. The increased PDCD5 expression in patients with hypothyroidism as well may reflect the enhanced apoptotic processes in these patients.

The last candidate gene, transcriptional regulator FOXP3 is an important determinant of Treg development and function (65). However, it is also highly expressed by the recently activated conventional human T cells (76, 77). Contrary to Tregs which stably express FOXP3, conventional human T cells transiently upregulate FOXP3 expression upon *in vitro* activation without acquiring suppressive capabilities. This happens also *in vivo*, especially in individuals with ongoing immune responses wherein T cells are chronically stimulated (78), and could explain the higher FOXP3 levels we found in our cohorts of patients. According to our results, upregulation of FOXP3 mRNA in peripheral T cells has been associated with HT, independent from the thyroid hormone status but proportional to disease activity (46). Transcriptional activity of FOXP3 is also dependent on its subcellular localization, which differs between Tregs and recently activated T cells, with a predominance in the nucleus for the former and in the cytoplasm for the latter cell population (79). Importantly, whether FOXP3 performs any additional effector functions in the cytoplasm which may affect patients still needs to be clarified.

According to these evidences, despite the finding of a FOXP3 overexpression in patients with MS, HT and MS + HT, we found decreased Treg levels in MS and HT compared with HC, with MS patients showing the lowest percentage. Tregs are a group of heterogeneous cells, with functional and phenotypic distinctions predicted by their cell surface marker profile. There is still no consensus regarding the best markers for human Tregs, thus the literature is not consistent with respect to the definitions of Treg subsets and their frequency and functions in both diseases (34). Nevertheless, a growing body of evidence, as well as recent findings by our group (43), suggest that these cells are numerically reduced and/or functionally impaired in MS patients. These cells might have a key role in the MS and HT pathogenesis influencing disease susceptibility and clinical course as well (35–42, 44). Interestingly, we found that Treg levels in MS + HT patients are similar to HC and significantly higher compared with MS patients. This result is difficult to interpret due to the paucity of literature in this field. Annunziata et al. described an inverse correlation between the titer of anti-Tg antibodies and the disability score in MS patients, suggesting a possible protective role of these antibodies that still needs to be further clarified (80).

Finally, 25-OH vitamin D is thought to modulate cell-mediated immune responses and to regulate inflammatory T-cell activity, shifting from a T-helper 1 to a Th2 phenotype and skewing away from the inflammatory Th17 phenotype. It has been shown to promote Treg development and functions as well (50–52). Our analysis revealed that the 25-OH vitamin D serum levels in MS patients are lower compared with both HC and HT, conforming to our findings on Treg levels. These data are also in line with the literature, as serum 25-OH vitamin D levels have been described to inversely correlate with the likelihood of relapse and disease activity on magnetic resonance imaging (81, 82), in addition to being associated with a better response to treatments such as interferon beta (83).

In conclusion, although very different from each other, MS and HT share certain common deregulated anti-inflammatory mechanisms, reflecting the autoimmune nature of both these disease conditions. However, there are some profound differences. MS patients are affected by a higher degree of deregulation of the analyzed factors compared with the HT group, which may indicate a more severe disease phenotype. HTE and HTI patients do not show relevant differences, apart from differential expression of genes implicated in the regulation of apoptosis. Patients affected by both diseases tend to have deregulations similar to the MS group, except for the Treg frequency, for which the co-occurrence of HT seems to reduce the defect.

These data, together with evidences from epidemiological and association studies, support the existence of shared deregulated mechanisms, that are represented here by BACH2/PDCD5-FOXP3 pathways and Tregs, involved in different extents across the spectrum of ADs. The biological implications of these findings need to be elucidated. Thus, further studies comparing different pathologies of autoimmune origin are vital to understand the core concepts of autoimmunity.

## Ethics Statement

This study was carried out in accordance with the recommendations of the Declaration of Helsinki. The protocol was approved by the local Ethics Committee of University Hospital San Luigi Gonzaga, Piedmont region (18/01/2013 N.20). All subjects gave written informed consent.

## Author Contributions

PS, BA, and OF developed the hypothesis and designed the project. PS supervised the research. PS, MS, MF, and GI conceived and designed the experiments. MS, GI, and OF contributed to the subject’s recruitment. GI, CS, MI, OF, and BA performed clinical examinations. PS, MS, MF, SM, and BG performed experiments. MS contributed to the statistical analyses. PS and MS wrote the manuscript. MF, GI, MI, PG, OF, and BA helped revised the manuscript.

## Conflict of Interest Statement

The authors declare that the research was conducted in the absence of any commercial or financial relationships that could be construed as a potential conflict of interest.
